# Striatal and extrastriatal dopamine transporter levels relate to cognition in Lewy body diseases: an ^11^C altropane positron emission tomography study

**DOI:** 10.1186/s13195-014-0052-7

**Published:** 2014-08-27

**Authors:** Marta Marquie, Joseph J Locascio, Dorene M Rentz, J Alex Becker, Trey Hedden, Keith A Johnson, John H Growdon, Stephen N Gomperts

**Affiliations:** 1 Department of Neurology, Massachusetts General Hospital, 15 Parkman St., Boston 02114, MA, USA; 2 MassGeneral Institute for Neurodegenerative Disease, 16th St., Building 114, Charlestown Navy Yard, Charlestown 02129, MA, USA; 3 Autonomous University of Barcelona, Medicine Doctoral Studies, Doctoral School, U Building, Autonomous University of Barcelona Campus, Bellaterra (Cerdanyola del Valles) 08193, Spain; 4 Department of Neurology, Brigham and Women’s Hospital, 221 Longwood Avenue, Boston 02115, MA, USA; 5 Department of Radiology, Massachusetts General Hospital, 55 Fruit St., Boston 02114, MA, USA; 6 Athinoula A. Martinos Center for Biomedical Imaging, 13th St., Building 149, Charlestown Navy Yard, Charlestown 02129, MA, USA

## Abstract

**Introduction:**

The biological basis of cognitive impairment in parkinsonian diseases is believed to be multifactorial. We investigated the contribution of dopamine deficiency to cognition in Parkinson disease (PD) and dementia with Lewy bodies (DLB) with dopamine transporter (DAT) imaging.

**Methods:**

We acquired ^11^C altropane PET, magnetic resonance imaging and cognitive testing in 19 nondemented subjects with PD, 10 DLB and 17 healthy control subjects (HCS). We analyzed DAT concentration in putamen, caudate, anterior cingulate (AC), orbitofrontal and prefrontal regions, using the Standardized Uptake Volume Ratio with partial volume correction, and we related DAT concentration and global cortical thickness to neuropsychological performance.

**Results:**

DAT concentration in putamen and in caudate were similar in PD and DLB groups and significantly lower than in HCS. Reduced caudate DAT concentration was associated with worse Clinical Dementia Rating Scale–sum of boxes (CDR-SB) scores and visuospatial skills in DLB but not in PD or HCS groups. Adjusting for putamen DAT concentration, as a measure of severity of motor disease, caudate DAT concentration was lower in DLB than in PD. Higher AC DAT concentration was associated with lower putamen DAT concentration in DLB and with higher putamen DAT concentration in PD. Higher AC DAT concentration in DLB correlated with greater impairment in semantic memory and language.

**Conclusions:**

Caudate and AC dopamine dysfunction contribute in opposing directions to cognitive impairment in DLB.

## Introduction

Neuropathological studies support multiple causative factors for cognitive impairment in Lewy body (LB) diseases. These factors include alpha-synuclein aggregation at synapses [1] and in cortical Lewy aggregates [2], amyloid deposition [3], and loss of the brain’s neuromodulators [4]. Dopamine plays a central role in the regulation of movement, reward-seeking behavior and cognition [5]. Dopamine neurons that innervate the putamen regulate movement selection [6], while those projecting to the ventral striatum, caudate, and cognitively eloquent cortices such as the cingulate cortex participate in reward-seeking behavior and cognition [7]-[10].

The dopamine transporter (DAT) is a reliable marker of dopamine neuron synapses in most brain regions. Localized to the presynaptic terminals of dopamine cells, the DAT terminates neurotransmission by reuptaking synaptically released dopamine [11]. DAT levels are high in the striatum and moderate in multiple cortical regions, including the anterior cingulate and the orbitofrontal cortex [8],[9].

DAT imaging via positron emission tomography (PET) or single-photon emission computed tomography permits its measurement *in vivo*. In LB disorders, striatal DAT levels reflect the dopamine concentration and fall as nigral dopamine neurons are damaged [12]. Whereas reduction in the putamen DAT concentration correlates with motor impairment [13],[14], reduction in caudate DAT relative to putamen DAT levels has been linked to cognitive impairment in dementia with Lewy bodies (DLB) [15],[16]. We explored these relations with altropane (2β-carbomethoxy-3β(4-fluorophenyl)-n-(1-iodoprop-1-en-3-yl) nortropane), which is a cocaine analog DAT ligand with fast kinetics [17]. Its high selectivity for the DAT over other monoamine transporters (dopamine/serotonin = 25:1, with minimal staining of the locus coeruleus) [18] makes altropane a specific marker of dopamine neurons, compared with β-CIT (dopamine/serotonin = 2.4:1) [19] or FP-CIT (dopamine/serotonin = 2.8:1) [20]. ^18^ F-DOPA, another dopamine imaging marker, has even lower specificity because it labels all cells expressing aromatic acid dopamine decarboxylase [21]-[23], which include dopamine, serotonin, and norepinephrine neurons.

In this study we measured ^11^C-altropane PET retention to assess the striatal and extrastriatal DAT concentration in cognitively normal Parkinson disease without dementia (PD) subjects, DLB subjects and healthy control subjects (HCS), and related regional DAT levels to cognitive function. We hypothesized that the putamen DAT concentration would distinguish the LB groups from HCS, and that, relative to putamen DAT, the caudate and extrastriatal DAT concentrations would fall in DLB subjects compared with the PD and HCS groups. We further hypothesized that the DAT concentration in the caudate, in midline and ventromedial frontal cortical regions, and in medial temporal regions would relate to cognitive function.

## Methods

### Participants

We enrolled 46 individuals, including 19 with PD and 10 with DLB. Seventeen HCS served as a control group. Subjects were recruited from the Massachusetts General Hospital Movement Disorders and Memory Disorders Units. They gave informed consent to participate in this research study according to the protocol approved by the Partners HealthCare Inc. Institutional Review Board. They underwent standardized neurological examination, cognitive testing, ^11^C-altropane PET imaging, and structural brain magnetic resonance imaging (MRI) for Freesurfer-derived partial volume correction of PET data. Cohort demographics, clinical features, and neuropsychological performance are presented in Table 1.

**Table 1:** Participant characteristics and neuropsychological performance

	**PD subjects**	**DLB subjects**	**HCS**
*n*	19	10	17
Age (years)	70.1 ± 7.0	72.1 ± 6.2	73.9 ± 6.7
Gender (female/male)	7/12^a^	1/9^b^	10/7^a^
Education (years)	16.4 ± 3.2	16.4 ± 3.5	16.2 ± 2.9
l-Dopamine equivalent dose (mg)	476.2 ± 379.8^c^	256.0 ± 280.3	0^d^
Hoehn and Yahr stage	2.5 ± 0.4^c^	2.25 ± 1.1^c^	0^e^
Unified Parkinson Disease Rating Scale	20.9 ± 7.4 ^f^	30.3 ± 2.9^b^	1.2 ± 2.5^e^
Duration of motor symptoms (years)	8.6 ± 5.1^a^	3.0 ± 0.7^d^	–
Mini-Mental State Examination	28.1 ± 2.4^a^	19.3 ± 9.2^b^	28.9 ± 1.5^a^
Clinical Dementia Rating Scale – sum of boxes	0.4 ± 0.6^a^	7.2 ± 3.7^b^	0.1 ± 0.2^a^
**Aggregate cognitive domain factors**			
Executive			
Trailmaking Test B (seconds)	112.4 ± 67.7^a^	298.8 ± 3.0^b^	73.3 ± 27.7^a^
Digit Symbol	41.3 ± 9.0^a^	14.1 ± 11.1^b^	47.8 ± 21.6^a^
Episodic memory			
Logical Memory I	12.9 ± 5.4^a^	5.6 ± 1.5^b^	16.5 ± 3.8^a^
Logical Memory II	12.1 ± 6.0^a^	5.4 ± 4.9^b^	16.1 ± 4.1^a^
Semantic memory and language			
Free Selective Reminding Test	28.8 ± 6.0^a^	18.4 ± 10.0^b^	32.9 ± 6.2^a^
Free and Cued Selective Reminding Test	47.0 ± 1.6	45.3 ± 2.6	46.3 ± 4.0
Boston Naming Test	27.7 ± 1.7^a^	23.1 ± 7.9^b^	27.9 ± 3.0^a^
Letter Fluency	44.9 ± 12.4^a^	24.0 ± 14.1^b^	52.7 ± 12.2^a^
Category Fluency	31.8 ± 6.9^a^	13.9 ± 9.5^b^	38.9 ± 15.0^a^
Visuospatial skills			
Visual Form Discrimination Test	28.9 ± 3.5^a^	24.7 ± 5.0^b^	30.6 ± 2.4

Values presented as mean ± standard deviation. PD, Parkinson disease without dementia; DLB, dementia with Lewy bodies; HCS, healthy control subjects. Significant differences (*P* < 0.05) between groups are indicated as follows: ^a^different from DLB; ^b^different from PD and HCS; ^c^different from HCS; ^d^different from PD; ^e^different from PD and DLB; and ^f^different from DLB and HCS.

PD subjects fulfilled diagnostic criteria for idiopathic PD [24] and were nondemented. The diagnosis of DLB was consistent with current consensus criteria [25]. HCS had normal neurological examinations, no cognitive complaints, a global Clinical Dementia Rating score of 0 [26],[27], and cognitive test scores in the normal range.

### Clinical and neuropsychological evaluations

Testing was acquired in the motor ‘on’ state to optimize cognitive performance [28]. The evaluation of motor function included the Hoehn and Yahr (H&Y) stage [29] and the motor subscale of the Unified Parkinson’s Disease Rating Scale (UPDRS) [30]. The following neuropsychological tests were administered: Mini-Mental State Examination (MMSE) [31], Logical Memory I and II (LogIA, LogIIA) [32], Free and Cued Selective Reminding Test (Free Recall, Cued Recall) [33], Letter Fluency [34], Category Fluency [35], 30-item Boston Naming Test [36], Digit Symbol component of the Wechsler Adult Intelligence Scale – Revised [37], Trailmaking Tests A and B [38], and the Visual Form Discrimination Test [34]. Functional status was assessed with the Clinical Dementia Rating – sum of boxes (CDR-SB) [26],[27]. Dopaminergic drug use was quantified as the l-dopamine equivalent dose (LED) [39]. We applied correlated factor analysis to the cognitive test performance of subjects to form four aggregate cognitive domain factors [40]: executive (Trailmaking Test B, Digit Symbol), episodic memory (Logical Memory I and II), semantic memory and language (Free Recall, Cued Recall, Boston Naming Test, Letter Fluency, Category Fluency), and visuospatial skills (Visual Form Discrimination Test). Cognitive domain factor scores were calculated as the average *z* score of the nonmissing component tests. A small number of subjects lacked some cognitive test scores, primarily due to dementia. No more than one-half of each factor’s component tests were allowed to be missing for a given subject.

### Imaging acquisition and analysis

Altropane was prepared onsite at the Nuclear Medicine Department at the Massachusetts General Hospital. PET images were acquired using an HR + PET camera (Siemens, Munich, Germany) operating in three-dimensional mode. After a transmission scan, 15 mCi of ^11^C-altropane was injected as a bolus and followed by a 60-minute dynamic acquisition. PET data were reconstructed and corrected for attenuation with vendor-provided software. Each frame was evaluated to verify adequate count statistics and absence of head motion.

MRI data (Siemens 3 T) were acquired using an MP-RAGE sequence. Freesurfer [41] (version 5.1 [42]) was used to align cortical folding patterns [43] and to parcellate the cortical surface and segment the subcortical grey matter into predefined regions of interest (ROIs). Each subject’s altropane data volume was mapped onto the Freesurfer-derived cortical surface in native MRI space by sampling the PET data at the midpoint of the gray-matter ribbon. ROIs included the putamen, caudate, thalamus, anterior cingulate (AC; formed by caudal and rostral AC), orbitofrontal (formed by lateral and medial orbitofrontal), prefrontal (formed by pars orbitalis, pars triangularis, rostral middle frontal, superior frontal and frontal pole) and medial temporal (formed by entorhinal, parahippocampus and hippocampus) areas [44]. Each subject’s altropane data were rigidly mapped to the MP-RAGE, and the resulting transformation was used to map the Freesurfer-derived ROI definitions (cortical gray-matter ribbon and subcortical gray matter) onto the native-space PET volume, in order to derive ROI DAT concentration regional averages.

The DAT concentration was estimated with specific binding of altropane, which was computed in ROIs using the standardized uptake value ratio [45], a ratio of uptake in the target ROI to the reference region measured between 40 and 60 minutes post injection. Pericalcarine (visual) cortex was selected as a reference on the basis of its low DAT concentration [9] and low altropane binding [17]. To compensate for the dilutional effect resulting from the low spatial resolution of PET, partial volume correction was applied to the altropane standardized uptake value ratio using the correction factor derived from the convolved binary brain mask (two-component Meltzer method) as described previously [46]. Global cortical thickness (GCT, mm) and the caudate volume (mm^3^) were derived from Freesurfer.

PET and MRI-derived measurements are presented in Table 2. DLB subjects showed a small (0.15 mm) but significant reduction in GCT compared with PD subjects (*P* = 0.003) but not compared with HCS. The caudate volume did not differ across diagnostic groups.

**Table 2:** Participant imaging data

	**PD subjects**	**DLB subjects**	**HCS**
Putamen DAT (SUVR-PV)	1.20 ± 0.18^a^	1.26 ± 0.38^a^	2.21 ± 0.46^b^
Caudate DAT	1.64 ± 0.35^a^	1.43 ± 0.48^a^	2.13 ± 0.46^b^
Anterior cingulate DAT	1.22 ± 0.12	1.12 ± 0.14	1.19 ± 0.12
Orbitofrontal DAT	1.26 ± 0.16	1.18 ± 0.16	1.27 ± 0.12
Prefrontal DAT	1.21 ± 0.21	1.02 ± 0.05	1.15 ± 0.12
Global cortical thickness (mm)	2.43 ± 0.12^c^	2.28 ± 0.12^d^	2.37 ± 0.08
Caudate volume (mm^3^)	3317 ± 424	3646 ± 647	3554 ± 665

Values presented as mean ± standard deviation. For the PET data, left–right averaged partial volume corrected SUVR values are shown. PD, Parkinson disease without dementia; DLB, dementia with Lewy bodies; HCS, healthy control subjects; DAT, dopamine transporter; SUVR-PV, standardized uptake value ratio partial volume corrected. Significant differences (*P* < 0.05) between groups are indicated as follows: ^a^different from HCS; ^b^different from PD and DLB; ^c^different from DLB; and ^d^different from PD.

### Data analysis

Group differences for demographic and neuropsychological measures were assessed with the analysis of variance test followed by the Tukey *post hoc* test for quantitative variables, and with the Fisher’s exact test for qualitative variables.

Medial temporal and thalamus ROIs were eliminated from the analysis because their group mean DAT concentration did not differ significantly from 1.0 according to three tests: a one-sample *t* test, and two nonparametric tests (sign test and Wilcoxon signed-rank test). The DAT concentration in the remaining ROIs was evaluated using a backward elimination general linear model (GLM) regressed on the initial predictor pool: diagnostic group, age, education, putamen DAT, duration of motor symptoms, LED and the interaction of diagnosis with each of the other predictors. The cutoff *P* value for removal from the model was 0.01. Note that we did not perform a multiple comparison analysis across ROIs. For HCS, measures of duration of motor symptoms and LED were set to 0 (with the addition of slight random normal perturbations).

We sought to use an imaging measure of motor disease severity in order to isolate it from cognitive performance [15],[16]. The putamen DAT concentration and its interaction with diagnosis were thus included as predictor terms when the putamen DAT concentration was not the dependent variable. The basis for this approach rests on the correlation of putamen DAT concentration with dopamine concentration [12], and on our observation that the putamen DAT concentration correlates strongly with UPDRS and H&Y scores in the whole cohort (UPDRS, *r*^2^ = 0.40, *P* < 0.0001; H&Y scores, *r*^2^ = 0.59, *P* < 0.0001), a finding shared by prior investigations [47],[48]. To touch base with previous imaging studies [47], we independently assessed the caudate/putamen DAT ratio. This is a less general approach than the GLM analysis.

A covariate of caudate volume and its interaction with diagnosis were also included when the caudate DAT concentration was the dependent variable. Tukey *post hoc* tests were performed as required to follow up significant diagnostic main effects.

We assessed the relation of the DAT concentration to cognitive test performance using GLM analyses with backward elimination (*P* > 0.01 for removal from the model), with a pool of predictors that initially included: diagnostic group, DAT concentration in the selected ROIs (excluding putamen), interaction of diagnosis with ROIs, GCT, caudate volume, age, education, duration of motor symptoms, and LED.

Distributions of residuals for all analyses were checked and verified as meeting test assumptions.

To exclude the possibility that the GCT covariate was redundant and multicollinear with DAT concentration, given that the altropane retention data were already partial volume corrected (using local rather than global volume), we reran all the analyses without including GCT in the GLM’s initial predictor pool, and the results were virtually unchanged.

SAS software (version 9.3; SAS Institute Inc., Cary, NC, USA) and JMP Pro software (version 10; SAS Institute Inc., Cary, NC, USA) were used for analysis and graphs.

## Results

### Subject characteristics

Diagnostic group differences in demographic, clinical, and cognitive variables are presented in Table 1. As expected, DLB subjects were more impaired than PD subjects and HCS on the CDR-SB (*P* < 0.0001), MMSE (*P* < 0.0001), and each cognitive domain factor: semantic memory and language (*P* < 0.001), episodic memory (*P* < 0.0001), executive function (*P* < 0.0001), and visuospatial skills (*P* = 0.0001). Cognitive scores in the PD and HCS groups did not differ significantly. Mean H&Y values were comparable between DLB and PD subjects (*P* = 0.50), and UPDRS scores were higher in DLB subjects than in PD subjects (*P* = 0.03). All DLB subjects were taking cholinesterase inhibitors when the PET scan, neurological examination and cognitive testing were performed, while none of the PD or HCS subjects were. Previous studies have demonstrated that treatment with cholinesterase inhibitors does not affect DAT uptake [49].

### Group differences in caudate and putamen dopamine transporter concentration

**Figure 1:**
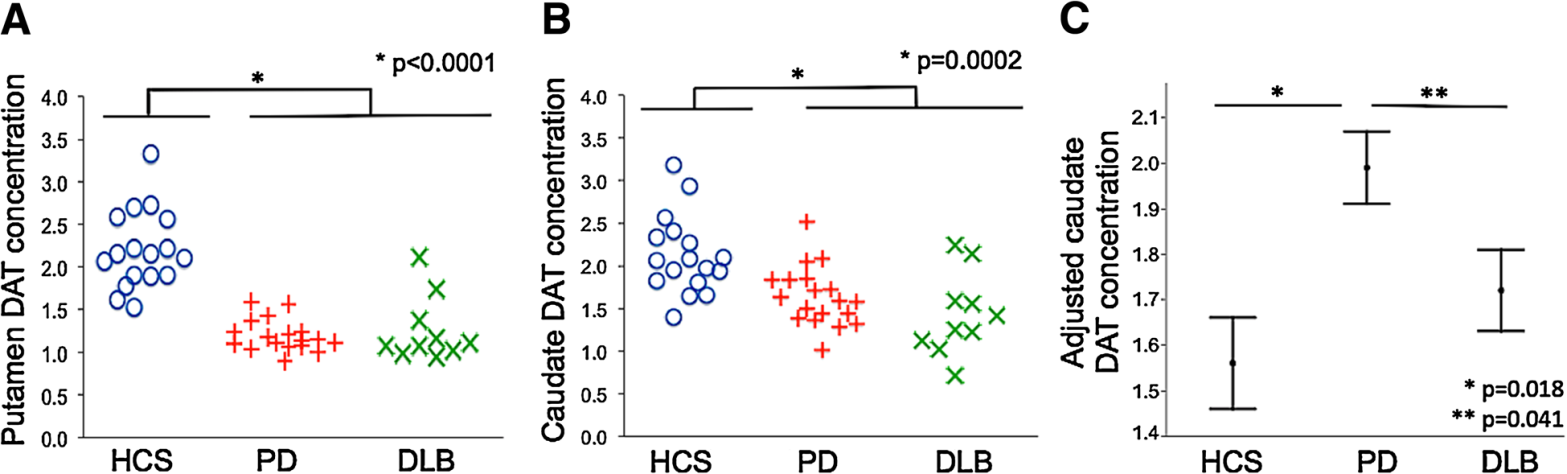
**Diagnostic group differences in putamen and caudate dopamine transporter concentrations. (A)** Unadjusted putamen dopamine transporter (DAT) concentration was similar in the PD and DLB groups, and was significantly lower than HCS (ANOVA, *P* < 0.0001). **(B)** Unadjusted caudate DAT concentration was also similar in PD and DLB subjects, and was significantly lower than HCS (ANOVA, *P* = 0.0002). **(C)** The adjusted caudate DAT concentration was significantly higher in the PD group than in the HCS (*P* = 0.018) and DLB (*P* = 0.041) groups. ANOVA, analysis of variance; DLB, dementia with Lewy bodies; HCS, healthy control subjects; PD, Parkinson disease without dementia.

Unadjusted DAT concentrations in the putamen and the caudate were similar in PD and DLB subjects, and significantly lower than in HCS (*P* < 0.0001 for putamen, *P* = 0.0002 for caudate, analyses of variance; Table 1, Figure 1A,B). The use of parkinsonian medications such as levodopa and dopamine agonists did not contribute to the regional DAT concentration. For the entire sample, lower putamen and caudate DAT concentration was associated with worse motor function (putamen: UPDRS, *r*^2^ = 0.40, *P* < 0.0001; H&Y, *r*^2^ = 0.59, *P* < 0.0001; caudate: UPDRS, *r*^2^ = 0.24, *P* = 0.023; H&Y, *r*^2^ = 0.24, *P* = 0.0005).

To study the relationship of the caudate DAT concentration with diagnosis independently of the severity of motor disease, we evaluated the caudate DAT concentration using a GLM that included putamen DAT concentration as a covariate in the original pool of predictors. This reduced to an analysis of covariance model, in which the parallel slopes assumption was satisfied for diagnostic groups (*P* = 0.43 for the test of difference in slopes). The adjusted caudate DAT concentration in the PD group was significantly higher than that for both the DLB (*P* = 0.041) and HCS (*P* = 0.018) groups (Figure 1C). The difference between PD and DLB subjects reflected a difference in relative caudate DAT binding, while the difference between PD subjects and HCS was driven by differences in putamen DAT levels. We found identical results when we used the caudate/putamen DAT ratio: a higher ratio in the PD group than in the DLB (*P* = 0.01) and HCS (*P* < 0.0001) groups (see Additional file 1).

### Group differences in cortical dopamine transporter concentration

We assessed group differences in DAT concentration in three cortical regions that subserve cognition: AC, orbitofrontal cortex and prefrontal cortex. Unadjusted DAT concentration in the AC was similar across groups (Table 2). Using a GLM model that related AC DAT concentration to putamen DAT concentration, global cortical thickness and other predictors (age, education, LED, duration of motor symptoms, and the interaction of diagnosis with each of the other predictors), we found that the diagnostic group and its interaction with putamen DAT concentration (*P* = 0.003) and with GCT (*P* = 0.008) was related to the AC DAT concentration (backward elimination GLM; for the model as a whole, *R*^2^ = 0.45, *P* = 0.003). Specifically, the relation of diagnostic group and AC DAT concentration was modified by the putamen DAT concentration, such that higher putamen DAT was associated with higher AC DAT concentration in the PD group, with lower AC DAT concentration in the DLB group, and with an essentially flat relation for the HCS group. The relation of diagnostic group and AC DAT concentration was also modified by GCT, such that higher GCT was associated with lower AC DAT concentration in the PD group, with higher AC DAT concentration in the HCS, and with a flat relation for the DLB group. These effects were limited to the AC, because the DAT concentration in the orbitofrontal and prefrontal ROIs did not differ between groups, even after accounting for differences in putamen DAT concentration and GCT (Table 2).

### Dopamine transporter concentration and cognitive performance

The diagnosis-dependent association of DAT concentration in the caudate and AC relative to DAT levels in the putamen led us to explore the relation of caudate and extrastriatal DAT concentration to cognitive function. For these analyses, we related regional DAT concentration to the CDR-SB, MMSE, and the four aggregate cognitive domain factors (see Data analysis).

**Figure 2:**
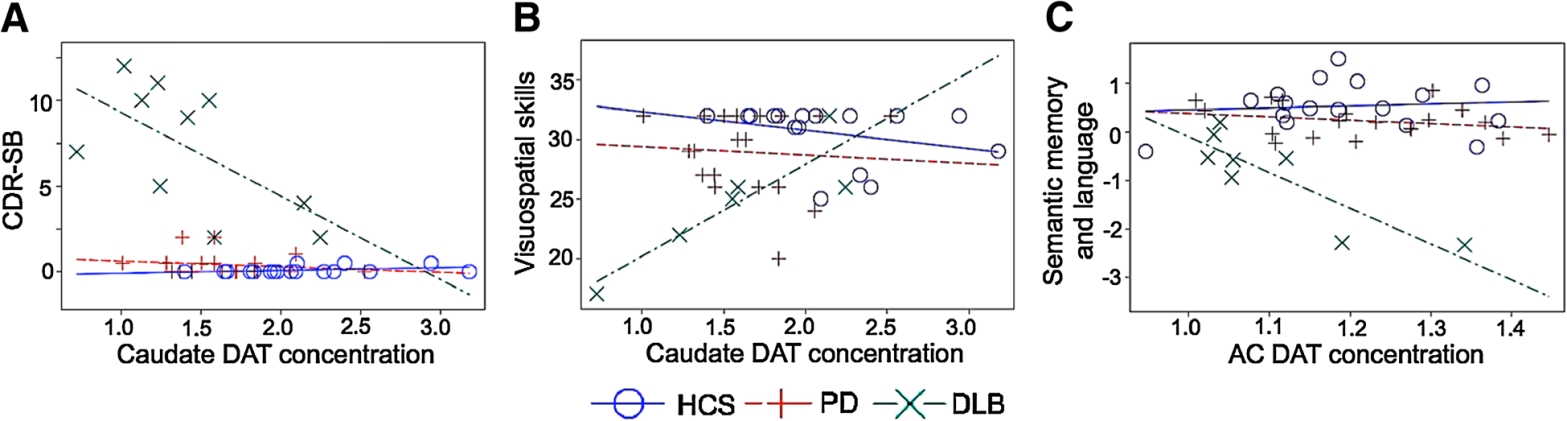
**Regional dopamine transporter concentration in the caudate and the anterior cingulate related to cognitive function.** In the DLB group, but not in the PD or HCS groups, loss of caudate dopamine transporter (DAT) concentration was associated with greater impairment in **(A)** CDR-SB scores (*R*^2^ = 0.84 and *P* < 0.0001 for overall model; *P* = 0.0008 for interaction between diagnosis and caudate DAT concentration) and **(B)** visuospatial skills scores (*R*^2^ = 0.45 and *P* = 0.0006 for overall model; *P* = 0.008 for interaction between diagnosis and caudate DAT concentration). **(C)** In the DLB group alone, higher anterior cingulate (AC) DAT concentration was associated with greater impairment in semantic memory and language performance (*R*^2^ = 0.69 and *P* < 0.0001 for overall model; *P* = 0.0003 for interaction between diagnosis and AC DAT concentration). Symbols indicate actual values; lines are predicted values from the general linear model. CDR-SB, Clinical Dementia Rating Scale – sum of boxes; DLB, dementia with Lewy bodies; HCS, healthy control subjects; PD, Parkinson disease without dementia.

We found in the DLB group alone that lower caudate DAT concentration was associated both with greater functional impairment, as measured with the CDR-SB (*R*^2^ = 0.84 and *P* < 0.0001 for model; *P* = 0.0008 for interaction between caudate DAT concentration and diagnosis; Figure 2A), and with greater visuospatial impairment (*R*^2^ = 0.45 and *P* = 0.0006 for model; *P* = 0.008 for interaction between caudate DAT concentration and diagnosis; Figure 2B). Further, in the DLB group alone, higher AC DAT concentration predicted greater impairment of semantic memory and language (*R*^2^ = 0.69 and *P* < 0.0001 for model; *P* = 0.0003 for interaction between AC DAT concentration and diagnosis; Figure 2C). The DAT concentration was not significantly associated with cognitive measures in either the PD or HCS groups. In contrast to DAT concentration, reduced GCT was associated with impairment in the MMSE (*R*^2^ = 0.53 and *P* < 0.0001 for model; *P* = 0.0035 for main effect of GTC) and in episodic memory (*R*^2^ = 0.50 and *P* < 0.0001 for model; *P* = 0.009 for main effect of GCT) in all diagnostic groups.

## Discussion

In this study we measured the regional DAT concentration with altropane PET and related the DAT values to diagnosis and cognitive function in nondemented PD subjects, DLB subjects and HCS. We found that both caudate DAT and AC DAT concentrations varied across the diagnostic groups, yet contributed to cognitive function in DLB in opposing ways: low caudate DAT levels and high AC DAT levels were associated with greater impairment on cognitive testing.

Consistent with prior reports [50],[51], putamen and caudate DAT concentrations were significantly reduced in LB disorders compared with HCS, independent of the presence of dementia. Although unadjusted caudate DAT concentration was comparable between PD and DLB subjects, as shown previously [52], the caudate DAT concentration adjusted for putamen DAT concentration as a measure of severity of motor disease was significantly higher in PD subjects compared with DLB subjects and HCS. These data confirm prior reports showing a higher caudate/putamen DAT ratio in nondemented PD compared with DLB [47]. The data suggest that when dopamine neurons that project to the putamen are damaged in LB disorders, a parallel injury of caudate-projecting dopamine neurons contributes to cognitive impairment. Although HCS had the highest unadjusted caudate DAT concentration, we also observed a smaller adjusted caudate DAT concentration in HCS compared with PD subjects, driven by the higher putamen DAT signal of HCS.

The AC DAT concentration related to the diagnostic group, after adjusting for putamen DAT, GCT and their interactions. Specifically in PD, the AC DAT concentration decreased as the putamen DAT concentration fell, whereas it was independent of putamen DAT levels in HCS. Surprisingly, however, in the DLB group the AC DAT concentration actually increased as the putamen DAT concentration fell. Thus, as dopamine cells die and putamen DAT levels fall into the range associated with parkinsonism, the pathological processes responsible for dementia affect how the AC DAT concentration changes.

The relations of caudate and AC DAT concentrations to cognitive test performance were consistent with the diagnostic group differences in regional DAT concentration. Loss of caudate DAT concentration in DLB subjects was associated with greater functional impairment, as measured by the CDR-SB, and with greater impairment of visuospatial skills. Although we are not aware of prior reports relating caudate DAT levels to visuospatial function, caudate dopamine levels have been associated with both executive function [15],[53],[54] and memory [55].

We also found that higher AC DAT concentration in DLB subjects was associated with greater impairment of semantic memory and language. To interpret this unexpected result, further research will be necessary to determine whether, in DLB, the DAT is a faithful marker of dopamine terminal density and dopamine concentration, or whether DAT levels and dopamine terminal density diverge. In this respect, striatal DAT levels in primates correlate tightly with striatal dopamine levels after 1-methyl-4-phenyl-1,2,3,6-tetrahydropyridine (MPTP) treatment [12],[56]. In addition, DAT levels transiently downregulate only very early in the course of PD, but do not appear to do so subsequently [57]. Lastly, we found no evidence for dynamic regulation of regional DAT levels as a function of the LED in our cohort, consistent with prior reports [57]. Thus, while it remains possible that AC DAT upregulation could occur in DLB, thereby reducing released dopamine to pathophysiological levels, we propose for now that AC DAT levels in DLB instead reflect dopamine terminal density and the local dopamine concentration.

**Figure 3:**
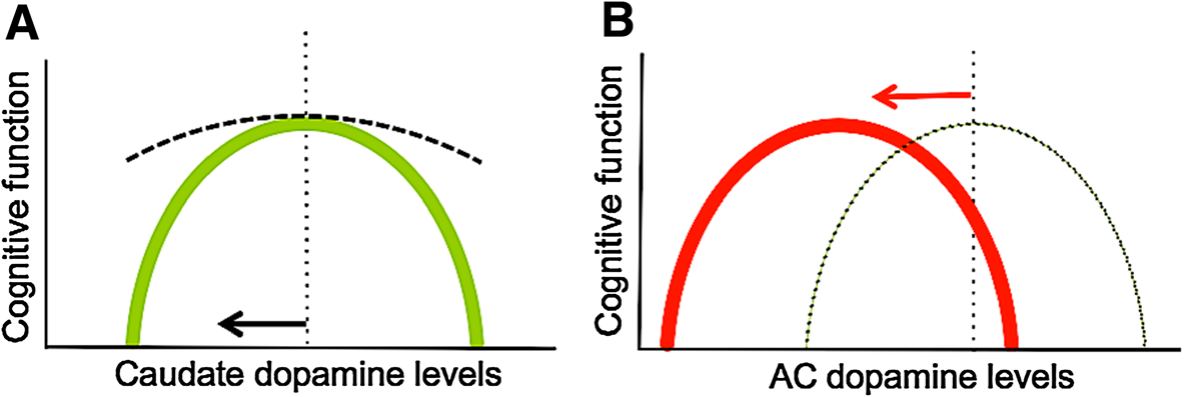
**Model to explain dopamine-associated cognitive impairment in dementia with Lewy bodies. (A)** In health, regional dopamine levels are maintained at a level optimum for cognitive performance (dashed line). In dementia with Lewy bodies (DLB), sensitivity to dopamine levels increases (green curve). Thus, as caudate dopamine levels fall, cognitive impairment worsens (arrow). **(B)** Anterior cingulate (AC) dopamine levels do not fall in DLB, yet they contribute to cognitive impairment. One explanation for this is that the inverted U-shaped curve moves to the left (red arrow and curve), such that previously optimized dopamine levels (dashed line) now contribute to cognitive impairment.

Prior preclinical and clinical work has demonstrated an inverted U-shaped relation between brain dopamine levels and cognition [58],[59], such that both excessive and insufficient dopamine levels impair cognitive performance. If pathological processes in DLB subjects increased the curvature of this relation in the caudate, this could explain how caudate and AC DAT levels in DLB subjects but not in PD subjects relate to cognition (Figure 3A). In addition, if pathological processes in the AC of DLB subjects shifted this inverted U-shaped curve to the left (or right; Figure 3B), previously optimum dopamine levels would now impair cognitive function and increases (or decreases) in dopamine would further worsen cognition.

Interestingly, cognitively normal PD subjects tolerated similar caudate and AC DAT levels as DLB subjects, without detectable impairment on cognitive testing. Dysfunction of the dopamine system therefore appears to contribute to cognitive impairment in DLB but is not sufficient for dementia. Other neuropathological factors, such as alpha-synuclein deposition [1], synaptic dysfunction [60], amyloid burden [40], and impairment of other neuromodulator systems [61], must make DLB patients susceptible to the impact of dopamine dysfunction.

Cortical DAT levels outside the AC did not distinguish between diagnostic groups or relate to cognitive test performance. This observation may reflect their differential innervation by midbrain dopamine cell groups [62]. Alternatively, the limited role for the DAT in dopamine clearance in some regions of the rat prefrontal cortex [63] may extend to humans. However, we cannot exclude the possibility that limited sample sizes may have obscured weak results. Future research with larger sample sizes will be useful.

Although cortical thickness was not the focus of this study, we also found that GCT was reduced in DLB compared with PD, and reduced GCT was associated with impaired MMSE and episodic memory performance. These observations are consistent with prior reports of cortical thinning in DLB [64].

The strengths of this study include the high specificity of altropane PET for DAT with negligible contamination from other monoamine transporters, accurate clinical diagnoses based on international clinical criteria, and the use of a comprehensive neuropsychological battery. An important technical note is the value of partial volume correction in altropane PET analyses, which enabled us to assess DAT levels not just in the striatum but also in cortical regions implicated in cognition.

## Conclusions

The results of this altropane PET imaging study suggest that dopamine dysfunction in the caudate and in the AC, along with cortical atrophy, contribute in opposing ways to cognitive impairment and dementia in LB disorders. To the extent that DAT levels reflect dopamine synapse density in DLB, the association of cognitive impairment with both low caudate DAT levels and high AC DAT levels may limit the potential benefits of a dopamine-related cognitive therapeutic for LB diseases.

**Note**

This article is part of a series on *Lewy Body Dementia*, edited by Ian McKeith and James Galvin. Other articles in this series can be found at http://alzres.com/series/LewyBodyDementia

## Abbreviations

AC: anterior cingulate

CDR-SB: Clinical Dementia Rating – sum of boxes

DAT: dopamine transporter

DLB: dementia with Lewy bodies

GCT: global cortical thickness

GLM: general linear model

H&Y: Hoehn and Yahr

HCS: healthy control subjects

LB: Lewy body

LED: l-dopamine equivalent dose

MMSE: Mini-Mental State Examination

MRI: magnetic resonance imaging

PD: Parkinson disease without dementia

PET: positron emission tomography

ROI: region of interest

UPDRS: Unified Parkinson’s Disease Rating Scale

## Competing interests

KAJ has received funding for travel and speaker honoraria from Pfizer Inc.; serves as a consultant for GEHC Ltd, Avid Radiopharmaceuticals, Inc./Eli Lilly and Company, Bayer Schering Pharma, Pfizer Inc, Elan Corporation/Janssen, and Bristol-Myers Squibb; and receives research support from Avid Radiopharmaceuticals, Inc./Eli Lilly and Company, Bristol-Myers Squibb, Janssen (Janssen AI), and Pfizer Inc. The remaining authors declare that they have no competing interests.

## Authors’ contributions

MM participated in the conception, organization and execution of the study, wrote the first draft, gave final approval of the version to be published, and agreed to be accountable for all aspects of the work. JJL designed and executed the statistical analysis, wrote the first draft, gave final approval of the version to be published, and agreed to be accountable for all aspect of the work. DMR contributed to acquisition, analysis and interpretation of the cognitive data, reviewed and critiqued the manuscript, gave final approval of the version to be published, and agreed to be accountable for all aspects of the work. JAB participated in the acquisition, analysis and interpretation of the image data, reviewed and critiqued the manuscript, gave final approval of the version to be published, and agreed to be accountable for all aspects of the work. TH participated in the analysis and interpretation of the image data, reviewed and critiqued the manuscript, gave final approval of the version to be published, and agreed to be accountable for all aspects of the work. KAJ participated in the conception, organization and execution of the study, reviewed and critiqued the manuscript, obtained funding for the study, supervised the project, gave final approval of the version to be published, and agreed to be accountable for all aspects of the work. JHG participated in the conception, organization and execution of the study, reviewed and critiqued the manuscript, obtained funding for the study, supervised the project, gave final approval of the version to be published, and agreed to be accountable for all aspects of the work. SNG participated in the conception, organization and execution of the study, wrote the first draft, obtained funding for the study, supervised the project, gave final approval of the version to be published, and agreed to be accountable for all aspects of the work. All authors read and approved the final version of the manuscript.

## Additional file

## Supplementary Material

Additional file 1: **Diagnostic group differences in the caudate/putamen DAT ratio.** The PD group showed a significantly higher caudate/putamen DAT ratio than both the DLB (*P* = 0.01) and HCS (*P* < 0.0001) groups. Values are mean ± standard deviation. HCS, healthy control subjects; PD, Parkinson disease without dementia; DLB, dementia with Lewy bodies; DAT, dopamine transporter. Click here for file
