# Supplementary figures and images for: Striatal and extrastriatal dopamine transporter levels relate to cognition in Lewy body diseases: an 11C altropane positron emission tomography study
Source: Alzheimers Res Ther. 2014 Aug 27;6(5):52. doi: 10.1186/s13195-014-0052-7 (PMC4245149; doi:10.1186/s13195-014-0052-7)

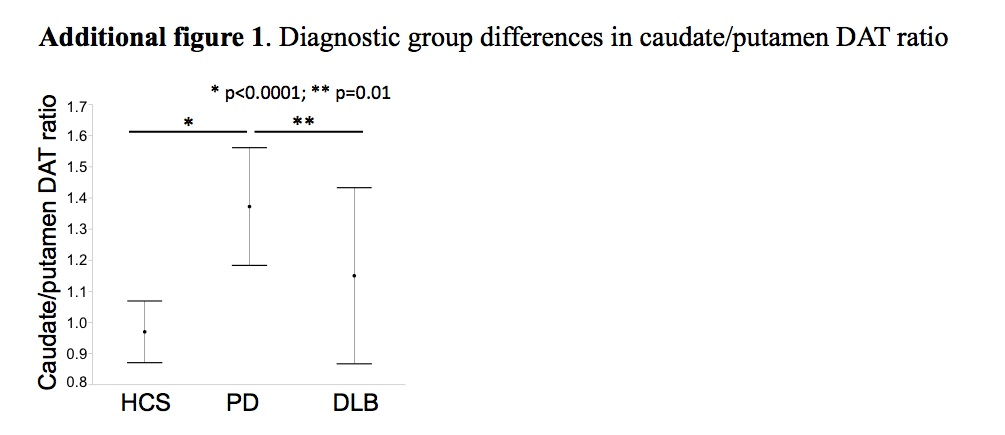

Supplement: Additional file 1: — Diagnostic group differences in the caudate/putamen DAT ratio. The PD group showed a significantly higher caudate/putamen DAT ratio than both the DLB (P = 0.01) and HCS (P < 0.0001) groups. Values are mean ± standard deviation. HCS, healthy control subjects; PD, Parkinson disease without dementia; DLB, dementia with Lewy bodies; DAT, dopamine transporter. [file s13195-014-0052-7-S1.tiff]
